# Parent Involvement in Mental Health Treatment for Autistic Children: A Grounded Theory-Informed Qualitative Analysis

**DOI:** 10.1007/s10578-023-01621-x

**Published:** 2023-10-17

**Authors:** Victoria Chan, Carly S. Albaum, Nazilla Khanlou, Henny Westra, Jonathan A. Weiss

**Affiliations:** 1 Department of Psychology, York University, 4700 Keele Street, Toronto, ON M3J 1P3 Canada; 2 Department of Psychiatry, The Hospital for Sick Children, Toronto, Canada; 3 School of Nursing, York University, Toronto, Canada

**Keywords:** Autism, CBT, Psychotherapy, Parent, Therapeutic Factors

## Abstract

Cognitive behavior therapy (CBT) is an effective treatment for many autistic children experiencing mental health problems, and parents are particularly involved in their psychotherapy. This study presents a conceptual framework of successful parent involvement in CBT for autistic children. Seventeen therapists (94% female) and 11 mothers were interviewed about their involvement in a CBT program for autistic children ages 8–13 years. The conceptual framework depicts how parent involvement varies depending on child, parent, and environmental factors. Parents’ contributions to therapy were grouped into five main roles. Parents’ beliefs and attitudes toward therapy also influenced their involvement. This is the first study to empirically investigate how parents of autistic children contribute to the therapeutic process in CBT.

**Supplementary Information:**

The online version contains supplementary material available at 10.1007/s10578-023-01621-x.

Cognitive behavior therapy (CBT) is an evidence-based treatment that addresses children’s mental health problems. The effectiveness of adapted CBT for autistic children is supported by randomized controlled trials, meta-analyses, and community-based research [1–3]. Some of the main ways CBT has been modified to better address the needs of autistic youth include using visual and concrete strategies, bringing in children’s specific interests, tailoring treatment to children’s needs, and increased levels of parent participation [4]. Indeed, parents are often involved in the psychotherapy that is provided to autistic children [5], varying from separate parent-only sessions to teach them about child activities [6], to having parents accompany their child during therapy sessions [7]. During child therapy sessions, parents may express their concerns about treatment, ask the therapist questions, and participate in activities [8].

Involving parents in CBT tends to lead to better child outcomes. For example, a recent meta-analysis of CBT trials involving autistic children found that interventions with parent involvement had greater reductions in child anxiety symptoms than interventions without parent involvement [9]. It is thought that parents might contribute to treatment outcomes by means of assisting with skill generalization [10] and by decreasing accommodation of children’s emotional or behavioral problems [11]. Although the specific mechanisms explaining the treatment benefits of parent involvement are unknown, the importance of incorporating parents in mental health treatment is widely acknowledged and a training program was recently developed for community mental health therapists focusing on increasing parent attendance in therapy sessions with autistic children [10]. In research involving neurotypical children with anxiety or obsessive-compulsive disorders, there is also preliminary support for treatments that focus on reducing family accommodation, which refers to the ways parents may change their behaviours to alleviate their child’s anxiety or distress (e.g., providing reassurance, modifying family routines, or allowing the child to avoid anxiety-provoking stimuli or situations) [11].

Relative to neurotypical peers, autistic children may benefit from higher levels of parent support in therapy settings due to the impacts of their social-communicative difficulties and associated neuropsychological challenges. Autistic children have social-communicative difficulties that can affect the formation of a strong working alliance with a therapist [12] and parents may help develop and maintain that alliance. Despite having similar physiological experiences of emotions compared to non-autistic peers, autistic children may also struggle with emotional insight or with expressing their feelings [13], which might affect their ability to identify physiological sensations and emotions in CBT. Challenges with executive functioning and cognitive flexibility [14] may also influence homework completion, and implementation of skills to different contexts and situations. To support some of these needs, parents may be asked to act as collaborators or “co-therapists,” help with homework completion [15, 16], model courageous behaviors, coach their child to use coping strategies [5], or discuss goals and teach skills through role-play [17]. Additional parent roles in the therapeutic process have been identified in the general paediatric psychotherapy literature, including: consultant (providing information to help determine the nature of the problem), co-client (if parents are contributing to or maintaining aspects of children’s difficulties), or collaborator (helping to implement treatment) [18]. Though there is evidence identifying the benefits of involving parents, there is a need to define parents’ roles in mental health treatment involving autistic children in order to better understand their influence on treatment outcomes.

Parent scaffolding may be a related component of their contribution to children’s therapy. Scaffolding refers to a parent’s support of their child’s emotional development in motivational and emotional ways [19]. Motivational scaffolding involves parents’ ability to initiate and sustain their child’s enthusiasm for a task, and may be shown through praise and encouragement, persistence, redirection of the child’s attention, or re-stating the goals of the task. Emotional scaffolding describes the parent’s ability to make the task a positive experience for the child, demonstrated by maintaining sensitivity towards the child’s emotions, sharing in the child’s positive emotions, and valuing the child’s participation. Parent scaffolding is associated with lower emotion dysregulation and/or fewer externalizing problems in neurotypical [19] and autistic children [20], and with improvements in toddler emotion regulation [21]. Of interest, the two forms of parent scaffolding (i.e., motivational and emotional) seem to map onto the two dimensions of therapeutic alliance (i.e., “task-related alliance” and “personal alliance” [22]), and emotional scaffolding incorporates other therapeutic factors such as empathy and genuineness.

There is also a need to identify the predictors and correlates of parent involvement in mental health treatment involving autistic children. Literature on parent engagement in other types of therapy (e.g., psychoeducational groups for parents of autistic children, autism early intervention) suggest that parents’ attitudinal and behavioral engagement in treatment are likely linked to intervention characteristics, relational factors, and external barriers or daily stresses [23], as well as parent self-efficacy, knowledge of autism and treatment, and belief in the therapy [24]. Though these findings have important clinical implications, they have yet to be considered within the context of autistic children’s psychotherapy.

## Current Study

This study aimed to elucidate relevant constructs and develop a conceptual framework to better understand the parent therapeutic factors at play in parent-involved CBT for autistic children. We conducted a qualitative study of therapist and parent perspectives to answer the question: *What does good parent involvement look like*? Parent-involved child mental health treatment was operationalized as therapy involving the presence of at least one parent or guardian for some or all of their child’s therapy sessions. Because the purpose of this research was to understand successful parent involvement in therapy, snowball and extreme case sampling [25] were used to identify parents who showed good examples of involvement in their children’s treatment. Parents were identified in two ways: (a) as nominated by therapists when asked to identify parents whose participation and involvement in sessions was thought to be particularly helpful, or (b) having a high degree of observed parent scaffolding prior to the start of the intervention. To identify parents with high levels of scaffolding, parent-child dyads were recorded in a standardized 15-minute discussion of emotional experiences, and parent scaffolding was coded using an observation rating system with known validity and reliability with autistic families [20, 21].

## Methods

### Participants

Participants included 17 therapists and 11 parents who were previously involved in one of two randomized trials of manualized CBT for children with neurodevelopmental disorders ages 8–13 years, called the *Secret Agent Society: Operation Regulation (SAS:OR)*. In the *SAS:OR* program, parents attended all 10 weekly sessions with their child, and were often involved in activities during and outside of therapy sessions. The first randomized controlled trial only included families of autistic children [26], and the second trial included families of children with a broader range of neurodevelopmental disorders. For the purposes of the present study, only parents of autistic children were involved.

Data collection and coding occurred in overlapping phases, and sample size was determined by theoretical saturation [27]. Of the 22 parents contacted through therapist nomination, 8 were willing and available to participate. An additional 12 parents were contacted based on their parent scaffolding, and 3 participated. Child treatment outcome was not considered when selecting parents to recruit for this study, and families who received direct therapy service from the first author were excluded to avoid potential bias effects. As shown in Table 1, most families involved male children (*M*_*age*_ = 9.18 years, *SD* = 1.54) and their mothers. Participants varied considerably in the time that elapsed since their last *SAS:OR* therapy session (2–55 months, *M* = 19.91, *SD* = 16.86). The 17 therapists were postdoctoral fellows or clinical psychology graduate students, and all except one were female. Therapists had completed *SAS:OR* with two to seven families (*M* = 3.65, *SD* = 1.45).

**Table 1:** Parent Demographics and Characteristics

	*M(SD)* or *%* (*n* = 11)
Age	
*Child*	9.18 (1.54)
*Parent*	43.27 (4.10)
Gender	
*Child (female)*	9%
*Parent (mothers)*	100%
Ethnicity	
*White*	73%
*Southeast Asian (e.g., Vietnamese, Cambodian*, *Malaysian, Laotian, etc.)*	18%
*West Asian (e.g., Iranian, Afghan, etc.)*	9%
Marital status (married)	82%
Graduated from college	91%
Family income (CAD before taxes)	
*< $49,999*	18%
*$50,000 - $99,999*	18%
*$100,000 - $149,999*	9%
*$150,000 or more*	27%
*Prefer not to disclose*	27%

*Note*: Data collected at time of participation in *SAS:OR* program

### Data Collection: Semi-Structured Interviews

The first author conducted all semi-structured interviews, and was involved in clinical trials of *SAS:OR* in a research and clinical capacity (evaluating and providing the intervention and supervising others). Participants answered initial open-ended, intermediate, and ending questions about parent therapeutic factors [28] (see Appendices A and B for interview guides). Open questions were broad in scope (e.g., “What was it like to be involved in your child’s therapy?”) to elicit participants’ immediate thoughts and ideas about the topic of interest. Intermediate questions (e.g., “What, if anything, was helpful about being involved in your child’s therapy? And what, if anything, was unhelpful or difficult about being involved?”) allowed participants to elaborate on their experiences and elicit more rich data.

### Procedure

Following approval from the institutional Research Ethics Board, therapists and parents were contacted to see if they were interested in participating in the interview. Participants provided written consent and could choose to participate at the university or their home. All participants were then shown a randomly selected video recording of one of their *SAS:OR* therapy sessions to facilitate their recall of their involvement in their child’s therapy (excluding the first and last sessions). During and immediately following each interview, the interviewer recorded field notes (i.e., personal thoughts and reactions to the data, and emerging possible relations between concepts [29]). Interviews were audio recorded and participants each received a $30 gift card at interview completion. The average length of interviews was 39.11 min (*SD* = 8.95, *Range* = 28–62), and interviews with therapists tended to be shorter in duration (*M* = 35.35 min, *SD* = 5.11, *Range* = 28–44) than interviews with parents (*M* = 44.91 min, *SD* = 10.66, *Range* = 29–62).

Using a grounded theory informed approach [27], recordings were transcribed verbatim by an independent transcriber, and using NVivo, coding occurred in three overlapping phases: open, axial, and selective. In open coding, coders assigned codes to capture the meaning of what was said. During this process, we applied analyst triangulation, where the primary and secondary coders coded several transcripts independently and met to compare their codes. After resolving coding differences, the primary coder carried out the remaining coding. Coders also used the constant comparative method, a process where phrases within each code were compared to one another to ensure they were conceptually similar to one another, while also being conceptually distinct from other codes. In axial coding, coders explored causal relationships between codes and grouped similar codes together under more general categories or concepts. Coding was driven by interview data, but coders were also sensitized to existing literature about parent involvement in children’s mental health treatment. Selective coding involved reviewing the reflective memos that were taken throughout data collection and analysis, which included the interviewer’s thoughts and reactions to the data, as well as emerging hypotheses [29]. Coders also examined relationships between categories and concepts by identifying confirming and disconfirming examples from interview transcripts. This process resulted in a conceptual framework, whereby categories and subcategories of codes emerging from interview data were causally related to one another.

### Methodological Rigor

Several grounded theory procedures were employed to enhance this study’s rigor and trustworthiness, including cyclical and overlapping phases of data collection and analysis, data-driven coding, grouping similar concepts into categories, applying constant comparison to ensure similarity of concepts within categories and distinctiveness between categories, and memo-ing throughout data collection and analysis. Analyst triangulation involved two independent coders meeting to consult on coding, and member checking involved presenting initial findings to a group of *SAS:OR* therapists and research staff to elicit their feedback, which was then incorporated into the conceptual framework.

## Results

**Fig. 1:**
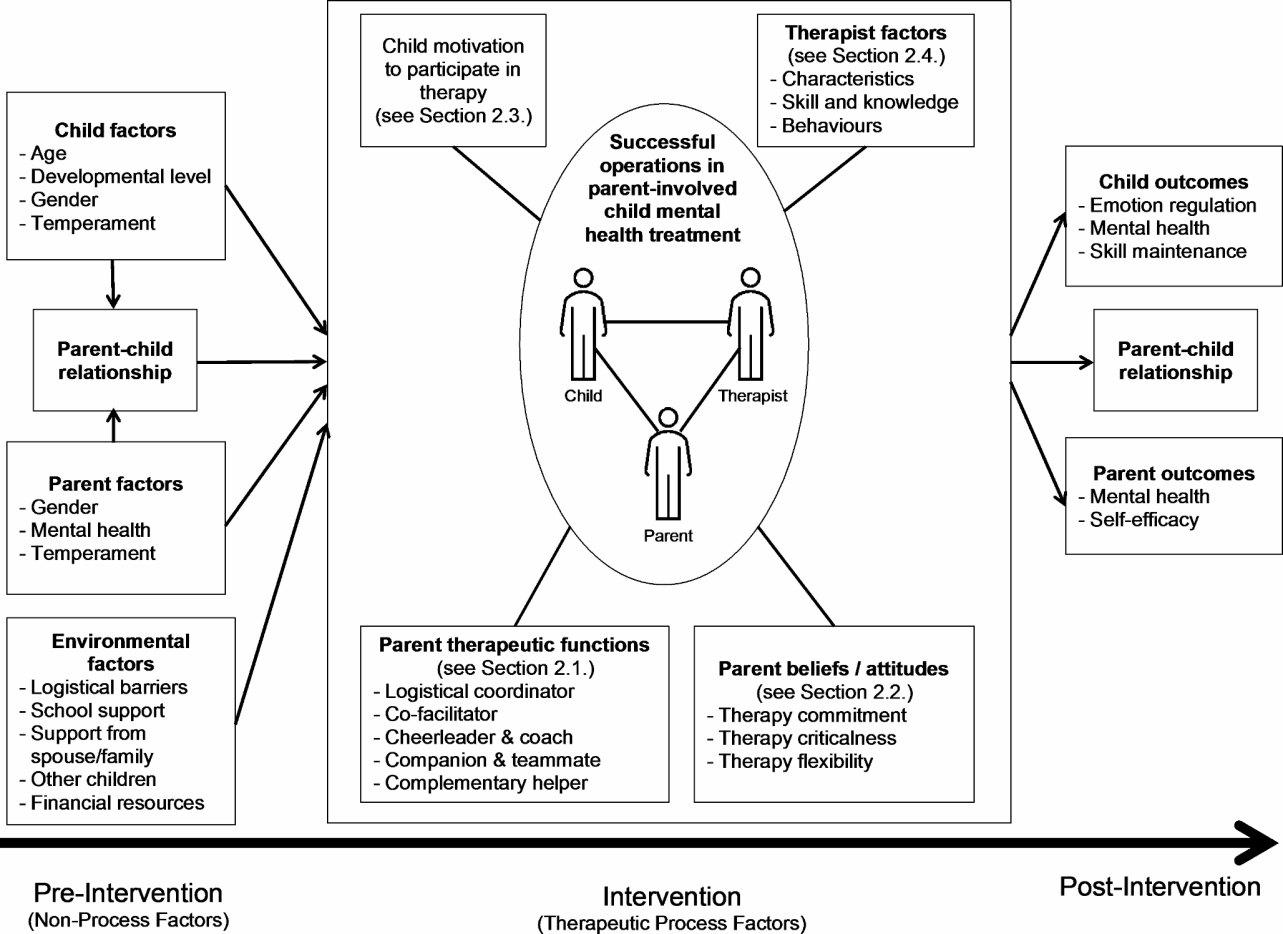
Conceptual framework of therapeutic process and non-process factors in parent-involved child mental health treatment

As shown in Fig. 1, a conceptual framework of parent involvement emerged from interview data and focused on four elements during the therapeutic process: parent therapeutic functions, parent beliefs and attitudes towards therapy, child motivation to participate in therapy, and therapist factors. These elements can be affected by pre-intervention factors, including child factors (e.g., age, developmental level, gender, temperament), parent factors (e.g., gender, mental health, temperament), the parent-child relationship, and environmental factors (e.g., logistical barriers, school support, support from spouse/family, other children, financial resources). In addition, therapeutic process elements may influence post-intervention factors (e.g., child outcomes, parent outcomes, and the parent-child relationship). Pre-intervention, intervention, and post-intervention factors are depicted linearly in the conceptual framework, and the focus of the current study is on the intervention (therapeutic process) factors (described in Section 2).

### Section 1: General Principles

There were three general principles that cut across the four elements of parent involvement: variability, goodness of fit, and the benefits of parent involvement in child mental health treatment.

#### Variability of Parent Involvement

Although all parents were seeking therapy for their children, not all parents demonstrated the same level of involvement in therapy activities. Therapists spoke of a range of parent involvement during sessions (e.g., participating in activities and discussions, attentive listening, following along in the parent handbook, helping the child understand therapy content), as well as outside sessions (e.g., facilitating skill practice at home, communicating with school staff).

Parents who were involved in a helpful way during therapy were noted as being *committed, motivated, positive, supportive*, and *mindful*. They demonstrated their *commitment* to therapy through completing in-session tasks and after-session homework, arriving to sessions on time, having therapy materials organized (e.g., bringing workbooks to and from home), implementing an at-home reward system for skill generalization, and being responsive to email or phone communication with the therapist outside of session time. Highly-involved helpful parents also tended to be *motivated* to engage with therapy content, were excited to attend sessions, eager to learn new skills alongside their child, were willing to try therapists’ ideas and suggestions, and were “immersed” in the program (e.g., learning the cognitive-behavioral language and using it at home). In addition, it was helpful when parents maintained a *positive attitude* towards therapy, demonstrated warmth towards their child, noticed and reflected on their child’s successes, and were *supportive* of their child’s progress. Helpful or involved parents demonstrated a level of *mindfulness* during sessions; they were present, “tuned in,” or “checked in to what was happening in the room,” and remained compassionate and non-judgmental during the therapeutic process.

There were also ways in which parents were unhelpful when included in treatment, such as being *disengaged* or *distracted*, frequently *interrupting*, or having a *negative attitude* or *misaligned session goals*. Therapists noted that some parents were *disengaged* or assumed that they were not needed in the session, and as a result could miss opportunities to step in and assist their child. There were also some therapy activities that participants noted seemed geared to the child alone (e.g., didactic psychoeducation, video games), which can send mixed messages given the program’s expectation to involve parents in all aspects of therapy sessions. While therapists understood that parents were busy and juggling multiple responsibilities, there were times when some parents used their phones, left the room, or were otherwise *distracted*, missing opportunities to support their child in activities. Therapists also found that parents who frequently *interrupted* their children or stepped in without letting their child speak first were unable to see what the child could achieve on their own. It was unhelpful to the therapeutic process when parents had a consistent *negative attitude* or affect in session, or when they had *misaligned session goals* leading them to be overly focused on details that took away from the session aim (e.g., making sure the child sat in the chair properly or spelled words correctly).

Of note, the *quality* of parental involvement seemed of greater importance than the *quantity* of involvement. For example, while some parents could be less talkative than other parents, they could still provide valuable contributions. Because of the many pre-intervention factors that can influence any given presentation of parent involvement, it may not be useful to try to describe an exact ‘rule’ or guideline as to how much or little parents should be involved in therapy.

#### Goodness of Fit of Parent Involvement

To fit with their child’s needs, parents adapted their involvement in therapy depending on child factors, such as age, developmental level (e.g., understanding of abstract concepts), executive functioning (e.g., organization, memory, problem solving skills), attention or behavioral problems, temperament, or motivation to engage in therapy. Participants noted how parents might not need or want to be present for the entire session if their child was older, capable of understanding the abstract concepts, or desired independent connection with therapists. In that case, parents could benefit from having the flexibility to attend the beginning and end of their child’s sessions, or to have some sessions without the parent to give the child some autonomy in the therapeutic process and the freedom to have their own ‘space’ with the therapist. Parents of children with executive functioning difficulties found they needed to provide their children with further support, such as breaking down tasks into smaller steps for their child, providing prompts and reminders to help support their children’s problem solving, help them stay organized, and plan home practice.

#### Benefits of Parent Involvement

Parent involvement was associated with benefits during and outside of session. During session, therapists noticed that when parents were actively engaged, children also seemed more motivated, had more fun, and were more likely to complete therapy homework. In effect, children learned from parents’ modelling of positive behaviors. In addition, learning and practicing emotion regulation strategies was challenging for many families, and the child’s persistence and motivation seemed to be bolstered when a parent supported their child through these challenges.

Attending sessions also had benefits that extended beyond session time. Parents learned content that could: (a) help them with their own emotion regulation and mental health, and (b) equip them to assist their child in emotion regulation tasks and in maintaining therapeutic gains post-treatment. For example, three years after participating in the brief CBT program, one parent (P008) was able to recall specific emotion regulation strategies they continue to use with their child, and described ways they adapted concepts learned in therapy to apply to more complex social situations as their child matured into adolescence. In addition, having a shared experience in therapy, along with a shared vocabulary to communicate about emotions and new tools to cope with strong feelings, can also enhance the parent-child bond. For example, one mother shared about how participating in CBT together helped to deepen her relationship with her son:

“I think it brought my son and I closer together, I think it really did. Being involved with him and walking every step of the way -- there was a deeper sense of trust I think with that. I became his sounding board for a lot of things, and I really credit this program, going through it all together, being involved in all of it. We were [close before] but it just took on a different element.” –P007.

Parents may also come away with a deeper understanding of, and compassion for, their child after participating in therapy with them:

“I think sometimes you don’t have the necessary tools to understand what that child is going through because you take for granted that simple things could be difficult. I saw how autism affected him, how challenging simple things were for him, and it made me parent differently. It made me feel stronger as an individual because I had more compassion for what he was going through.” –P011.

### Section 2: Therapeutic Process Factors

Four therapeutic process factors were seen as important elements of parent involvement (components “in the room” during therapy), including parent therapeutic functions during the intervention (e.g., logistical coordinator, co-facilitator, coach and cheerleader, companion and teammate, complementary helper), parent beliefs and attitudes towards child mental health treatment (e.g., therapy commitment, criticalness, and flexibility), child motivation to participate in therapy, and therapist factors (e.g., characteristics, skill and knowledge, behaviors).

#### Parent Therapeutic Functions

Parent-specific therapeutic process factors included five therapeutic functions parents may fulfil during the intervention: logistical coordinator, co-facilitator, coach and cheerleader, companion and teammate, and complementary helper.

##### Logistical Coordinator: “Let me handle the logistics”

Parents were in charge of planning and organizing the logistics involved with attending sessions. This involved communicating with the therapist to schedule sessions, arranging transportation, ensuring that materials (e.g., handbooks, worksheets, cards) were brought to and from home, coordinating with school staff if session times overlapped with the school day, bringing snacks, and allotting time for traffic to arrive on time. For example, one therapist said:

“I think that it was really nice to have the parents there because they would be better [than the child would be on their own] at organizing all these materials and making sure things are brought to session, not forgetting things. So that management aspect of it was very helpful. Again more logistics stuff but it makes a big difference actually, especially with a CBT program.” – T016.

##### Co-facilitator: “Here is what we need to do”

During sessions, parents assisted therapists with the execution of therapy tasks and were engaged in co-running aspects of the intervention with the therapist. Working as a team, parents brought in expertise on their child (e.g., providing the therapist with relevant clinical information about the child’s history and interests), while the therapist brought in their expertise on the therapy content with the parent helping to facilitate activities (e.g., helping the child understand how to do therapy activities). One therapist (T005) noted: “Just working together, they know their kid better than me, so they know if I word something one way, they might know how to word it differently to make their child understand.” CBT often relies on clients to provide examples of a time when they felt a certain emotion to help guide discussions during therapy, and therapists found it helpful to have parents remind their child of specific examples from the child’s experiences at school or at home.

Parents also provided therapists with insight into the child’s life, articulating recent challenges that may have arisen, and providing therapists with feedback on the implementation of skill practice at home. They also gave therapists an idea of when a particular topic might be difficult for the child. For example, one therapist shared:

“…the parent would lean over and something would be said and they’d [say] ‘Oh this is a tough topic for him, this will be hard today.’ And they give you this little window [into the child’s life] that you wouldn’t probably get without the parent.” – T005.

Parents can also provide therapists with helpful information about a child’s strengths and interests, enabling therapists to incorporate those into therapy. In turn, parents’ co-facilitation efforts help to make the material more relatable to a particular child’s context. Over the course of therapy, as parents learned how to help their child identify and rate emotions, and implement coping strategies, some parents spontaneously drew on these skills in-session to support their child during times of emotion dysregulation, with the assistance of the therapist if needed.

Parents were also co-facilitators *outside* of the session. For example, parents were instrumental in ensuring weekly completion of home practice, orchestrating situations to enable their child to practice the emotion regulation skills, reminding the child to complete home practice, creating an at-home reinforcement system similar to the one used in session, and communicating session content to school staff to help the child practice skills at school. Some parents also found creative ways to complete home practice (e.g., watching the Olympics to practice identifying others’ emotions through facial expressions or body language). Because parents observed how therapists explained concepts in session and learned therapy content first-hand, parents were able to use consistent terminology and language with their child, as well as engage in problem-solving when barriers or challenges arose during skill practice at home or at school.

##### Cheerleader and Coach: “You can do it and I can help you succeed”

Parents encouraged children in targeted change (i.e., improving their emotion regulation skills), with the goal of increasing the child’s autonomy in coping with their emotions. Parents provided their child with praise and encouragement for completing difficult activities or doing their best to learn new concepts or skills, as well as celebrated and delighted in the child’s successes, such as one parent who said:

“Like when [my son] worked through that anxiety too by himself and I’m just coaching from the sidelines, like giving him extra points, he just—wow! So then [I’d say]… ‘there are times you didn’t realize how big this is that you did this! You did this, right? Yourself! This is huge!’” – P007.

If child motivation waned towards the later stages of therapy, parents would also encourage their child to continue. There were times when coping strategies would no longer work, or when families had to go through a period of trial-and-error to identify new strategies, and in those times, parents helped to keep their child moving toward therapy goals. One mother talked about drawing on her personal experiences of the effectiveness of coping strategies to encourage her child to continue practicing these new skills, even when it seemed difficult:

“I think because I could relate and I could see how certain concepts of, for example deep breathing, are so good, I knew that they could work with him as well […] I could encourage him to persevere because if you’ve been through a tunnel you’ve come out the other side, you know there is another side, right? So then you can help him through that as well.” -P007.

It was also important to parents that they balance supporting their child with allowing them space to learn how to use coping skills and regulate emotions more independently. Parents felt that providing praise and encouragement also contributed to their child’s confidence and sense of accomplishment. One mother spoke of how rewarding it was to be part of her child’s progress:

“…it was kind of fun, you feel a little bit [like] a mother hen pushing her child out a little bit. But it was so wonderful to see to see him grow, and to be able to pull back and on the sidelines and just kind of push him a little bit out there saying, ‘You can do this, I’m watching you do it.’” – P007.

##### Companion and Teammate: “I am here for you, we are in this together”

Parents were able to act as children’s allies and build a sense of “togetherness.” At the beginning of therapy, a parent’s presence can help them feel more comfortable with the novel environment, making it easier for the child to see therapy as a safe space and enable the therapist to build rapport. Parents were also involved alongside their children in activities, such as role-play to practice coping skills and problem solving, or game-based psychoeducational activities. By being a companion with the child, parents provided a sense of companionship and normalized the experience of receiving help from a mental health professional and allowed the child to feel less alone in the process, knowing that they had someone to trust and rely on. Some parents also found ways to involve other family members in home practice or to teach their other children coping skills to normalize the use of these skills.

Having the parent and child together during sessions allowed them the opportunity to work as a team. One therapist (T016) recounted an example where, “They were on a team and there was a lot of support and a lot of trust, and I think it was a real amazing asset to have them together because it elevated it to another level.” Some parents were enthusiastic about participating to help their child get the most out of their therapy experience, with one therapist (T005) remarking, “I had parents on the ground, making jokes with their kids but keeping the kid comfortable. I had some great examples of parents who would really do anything to get their child engaged and going through the program.”

##### Complementary Helper: “I can fill in the gaps where you need me to”

This function specifically refers to the acts of helping to bridge the gap between the child’s capacity and what is expected of them within the therapeutic context. By being attuned to the child’s needs, parents could step in to scaffold a child’s participation in session through actions such as: helping the child build a relationship with the therapist, helping the therapist better understand the child, managing the child’s behavioral or attention problems, scribing for a child with fine motor issues, or linking therapy content to the child’s experiences to make abstract concepts more concrete and understandable. For autistic children with social-communication difficulties, developing a relationship with an unfamiliar person can be challenging, and some autistic children may need their parent’s help with building a therapeutic relationship with the therapist. Parents can “bridge the relationship” between the therapist and the child by modelling for the therapist ways to best to relate to the child, especially when a child is less intrinsically motivated to engage with the therapist. Some parents helped their child develop a rapport by prompting them, or by offering information about the therapist that would pique the child’s interest:

“the mom said to [her son], “Did you know that [therapist’s name] has her PhD?” And he like, looked away and then two seconds later, he looked up, he’s like, “Really? What do you have it in?” And all of a sudden he was interested in me… she knew what he would like about me and she tried to develop that relationship.” –T008.

Parent complementary helpers also bridge the gap for children by asking therapists to change aspects of sessions to better accommodate specific triggers or needs (e.g., noting how a child can be triggered when hungry can lead to incorporating snack breaks into session agendas). Parents used strategies to help support their child when faced with attentional issues, such as repeating or rewording a question, reminding the child to stay focused, and redirecting the child’s attention back to the goal of the session. In other instances, children experienced negative emotions that could have led to a rupture in the therapeutic alliance and parents functioned as a ‘safety net’ in these situations. One therapist (T008) noted: “I can do my best but [the mother is] the one that gives him a little whisper in the ear. I don’t know what she’s saying but then he starts to actually listen to me.” Children who had fine motor issues benefited from having a parent scribe for them when they needed to complete written activities. CBT also requires a certain level of abstract thinking, as clients are asked to change or bring awareness to maladaptive thoughts. Parents were connecting the dots with their child and helped them understand how to apply a coping strategy by reminding them of a situation in which it might have been useful, or bringing in other personal examples to “bring the material to life for the child” (T017).

#### Parent Beliefs About and Attitudes Towards Therapy

In addition to these parent therapeutic functions, three main parent beliefs and attitudes related to therapy emerged as important in influencing parent involvement: therapy commitment, therapy criticalness, and therapy flexibility.

##### Therapy Commitment: “Therapy is worthwhile,” Positivity, and Persistence

Many parents were committed to therapy, demonstrating an attitude of positivity and persistence, and the belief that participation in therapy is worthwhile. Therapists noticed that some parents seemed particularly committed to prioritizing therapy in their busy schedules, taking an active role in completing therapy tasks (e.g., in-session activities, practicing coping strategies with the child at home), arriving to sessions on time, and a general ‘buy-in’ to the effectiveness of therapy. Parents also understood that helping their child outside of session time and after the CBT program ended would help their family get the most benefit out of therapy: “It’s only as good as the effort you put in after the fact, so [I was] trying to keep drawing on the material and keep it fresh at home” (P005).

Parents also reframed potential barriers to therapy in a positive light. For example, one mother reframed long travel times as an opportunity to bond with her child. Another parent described their commitment to their child’s therapy as a small part of a bigger picture:

“I guess the more you learn about something like autism and how that’s going to affect your child… how it shows in their life, you know, it’s a journey. …this is going to be a journey and [SAS:OR] is then part of the journey.” – P007.

Therapists reported that it was helpful to have parents look for positives, even when things were hard in sessions; and to have parents be excited to participate and supportive of the overall process, without expecting every strategy to work flawlessly.

##### Therapy Criticalness: “Therapy is an added stressor”

The term ‘criticalness’ is sometimes used in a negative tone, but here it is used neutrally to capture a posture of thinking critically about the demands that participating in therapy place on a family and available resources. For example, one parent appreciated how her presence in therapy sessions was helpful for her son, but also felt she could have benefited from an hour’s respite to prevent caregiver burnout:

“I wanted to be a part of [therapy] so I could really capture what they were doing and what was helpful. If I’d been sitting in the waiting room, I wouldn’t have gotten that full effect, which I think is really good. But I did find it, to be honest, exhausting, just because it was a long drive there and because he was a bit resistant to go. We really want to help our kids too but there’s also never time for us either, and then we get burnt out.” –P010.

Even if parents think that their involvement in their child’s CBT is helpful, they also benefit from setting realistic expectations of how much their family is able to invest in therapy, understanding that each family is approaching treatment within a different context. Therapists can model this for parents by being flexible and decreasing the pressure to complete prescribed homework.

##### Therapy Flexibility: Flexibility, Openness, and Letting Things Go

Many involved parents were flexible in their implementation of CBT strategies, were open to trying new ways of coping, and were willing to let things go when situations did not go as planned. Having learned the therapy material alongside their child, parents were empowered to adjust coping strategies as required by different situations, by children’s changing interests, or by different maturity levels:

“It’s like a dial on an instrument and every time his interest or his maturity level [changes], depending on the season, depending if there’s stress factors in his life, you have to turn the dial and adjust the methods that you use. Now I have to use this method a little bit more, this one a little bit less. It’s not working in this situation and so forth. […] And then use the experiences I have from SAS or other strategies from the past and try to merge them and try to come up with a solution.” -P002.

Part of flexibility within a therapeutic context is having openness, whether it is trying new things, managing child emotions in a different way, or giving a strategy another chance. Therapy might be a new experience for both the child and the parent, and it was helpful for parents to bring openness to their experiences and those of their child. Having openness does not necessarily mean that all new experiences will be positive; some parents learned to let things go when they encountered difficulties. Some parents had an adaptive response to managing multiple competing demands by prioritizing their focus and by granting themselves compassion to focus on what they could handle in the present moment.

#### Child Motivation

Parents and therapists both noted a reciprocal process of child motivation and parent involvement. When children came to their first therapy session with high intrinsic motivation, parents did not need to be as involved because there was less of a need for parents to model positive behaviors to maintain their child’s engagement. When children had low motivation to attend therapy, considerable parental effort was needed:

“He would just move slow[ly] […] when we got to the parking lot, and even just getting out of the parking lot once we got there. So I would do mini-steps, like ‘Well, you know if we just get from here to the elevator then we’ll all talk about it some more.’” – P010.

Therapists also noticed that when parents were actively engaged, children also seemed more motivated, had more fun, and were more likely to continue completing therapy homework, even as families faced increasingly difficult challenges as part of the therapy program. For example, a therapist recalled:

“…[when] they would try to use a [coping skill] and it didn’t go so well or [when] they tried something that was really difficult, [the parent] always found a way to be really positive and supportive and find the strengths in those moments. [The parent] was really proud. And the child really picked up on that, and that really motivated him to keep going and trying” –T009.

#### Therapist Factors

There were also key therapist factors that were associated with parent involvement: therapist characteristics and behaviors, and their level of skill and knowledge. Parents found it helpful when therapists showed compassion and empathy, warmth, eagerness, and flexibility. One mother said:

“…whether it’s SAS or any other therapies I’ve gone to, I always feel like I sit in front of a professional who gives me great advice, but just doesn’t understand the emotional components of how exhausting all this is […] those levels of compassion are so important.” –P003.

Therapists appeared to support involvement through specific behaviors. Early in therapy, therapists noted how they clarified what therapy would look like, and expectations during and outside sessions. They also noted that it was helpful to ask parents if there were any barriers to involvement, gauging treatment readiness, and problem solving with parents to set them up for success. Parents and therapists described the importance of therapists making an effort to learn more about families’ previous therapy experiences, asking about what did or did not work from week-to-week, checking-in on parents’ understanding, and aiding parents’ knowledge of how to support their child in therapy.

Many therapists in this study were graduate-level trainees, and those with less experience voiced worries about the possibility of the parent evaluating them negatively, as well as concerns about overstepping parents’ authority or about setting boundaries if parent-child conflict arose. Beginner therapists also appreciated having the parent present for sessions because it helped them with managing child attentional or behavioral problems, and with learning more about the child (e.g., when parents filled complementary helper and co-facilitator functions). Therapists needed to be attuned to both the child and the parent in session and had to build a therapeutic alliance with both, and noted managing two therapeutic relationships by emphasizing collaboration and teamwork among the three parties.

## Discussion

This study is the first qualitative study of how parents of autistic children contribute to the therapeutic process in parent-involved child psychotherapy. There is evidence that CBT trials with parental involvement tend to have greater treatment effect sizes (i.e., greater reductions in autistic children’s anxiety symptoms) compared to CBT trials without parental involvement [9], and parent involvement may also lead to improvements in parent mental health [30], but there is a paucity of research on the specific ways in which parent involvement might be helpful in CBT for autistic children. Participation in CBT involves a client’s ability to identify their thoughts and emotions, and to form a therapeutic alliance within a short time frame [31], requiring verbal expression, sustained attention during didactic teaching, identification of cognitive processes, and emotional awareness, all of which can be particularly challenging for autistic children [13, 14], making parent involvement a critical consideration for treatment accessibility.

This study’s results place an emphasis on the goodness-of-fit of parent-child interactions in the context of therapy. Parents often naturally adapted their interactions with their child during therapy depending on the child’s need for support and the situational demands. This can include a balance of filling more ‘child-led’ parent therapeutic functions (e.g., coach and cheerleader, companion and teammate) with more ‘parent-initiated’ therapeutic functions (e.g., logistical coordinator, co-facilitator). Parent responsivity is found in theoretical models of transactional relations between self- and other-regulation in child development [32], in longitudinal research on parenting stress and behavior problems of children with developmental disabilities [33], and in other forms of parent-involved child treatment. For example, in Parent-Child Interaction Therapy, parents are given direct coaching on how to follow the child’s lead in *child-directed interactions* (using positive parent strategies such as positive attention and praise) and to initiate *parent-directed interactions* (using straightforward commands) when necessary to decrease inappropriate child behaviors [34]. The conceptual framework is a bioecological model of a number of pre-intervention (non-process) and intervention (therapeutic process) factors. Also referred to as the Process-Person-Context-Time model [35], successful operations are influenced by *person* characteristics (e.g., child and parent characteristics) and the family’s environmental *contexts* (e.g., immediate and extended family, school, financial resources), which may change over *time* (e.g., changes pre- to post-intervention).

### Parent Therapeutic Functions

This study goes beyond previous research by articulating five specific therapeutic functions that parent involvement in CBT might serve: *logistical coordinator, co-facilitator, coach and cheerleader, companion and teammate*, and *complementary helper*. These functions are not hierarchical, as no one therapeutic function seems to serve as a precursor or prerequisite to another. A parent may fill each therapeutic function to varying degrees, depending on their ability to serve each function, their child’s relative need for support in that way, and their family’s context. In other words, ‘ideal’ parent involvement in CBT could be a different combination of therapeutic functions depending on the skills and characteristics parents come in with, as well as the needs of the child and the family’s context.

The importance of *logistical coordinator* should not be underestimated, particularly for parents of children with complex needs who might also be juggling appointments with other health care providers, child care for multiple children, or other stressors on top of work demands or transportation limitations. Parenting stress tends to be high for caregivers of autistic children [36], but the relation between parenting stress and negative outcomes (e.g., depression, social isolation) can be moderated by positive ways of coping (e.g., positive reappraisal, taking action [37]); and one example of a parent’s positive coping might be seen in organizing and coordinating the logistics involved in therapy participation. The *co-facilitator* function can help with skill generalization to other contexts outside of session, and maintain therapeutic gains through parent-facilitated skill practice after therapy ends [5]; this can contribute to maximizing a child’s benefit from a brief intervention, such as the 10-week CBT program employed with this study’s participants. Parents acting as co-facilitators can also increase the ‘dosage’ of intervention the child receives, similar to the use of parent-mediated intervention in early intervention programs for younger children with autism [38], as parents continue to help their children consolidate their understanding of therapy content outside of weekly one-hour therapy sessions.

The *coach and cheerleader* function is similar to scaffolding, whereby parents initiate and maintain their child’s enthusiasm to complete a task, and make the task a positive experience for the child [19]. Coaching in sessions may also capitalize on the ‘zone of proximal development,’ enabling the child to achieve more of their potential than they would without support [39]. Supporting children in therapy also involves being a *companion and teammate*, with parents acting as an ally with the child, building “togetherness.” Similar to therapist self-disclosure, which can normalize challenges a client might face in the therapeutic process [40], parents’ ability to model the effectiveness of coping strategies and share in the highs and lows of therapy can motivate children to persist and maintain enthusiasm. The parent-child team allows the therapist to form a triadic relationship, similar to couple’s therapy, where the therapist can work with the two clients as a unit (where the therapist’s focus is on the clients’ relationship together) or as a translator (providing each client with empathic understanding and mediating interactions to help clients enact a new way a relating to each other), instead of forming bonds with each client separately [41].

The last function, being a *complementary helper*, is when the parent partners with the child and therapist to bridge the gap between the child’s capacity and what is expected of them, increasing therapeutic accessibility for children with different strengths and needs. It was helpful for parents to complement children’s autism-specific needs (e.g., socio-communication skills needed to build a relationship with the therapist) and needs common among neurodevelopmental disorders more broadly (e.g., attention or behavior problems, organization and memory challenges, fine motor issues, difficulty understanding abstract concepts). Systemic approaches to family therapy describe complementary relationships as a way of relating based on difference or opposites, such as one person being cared for and the other person providing their care [42]. Although complementary relationships can be problematic when dyads are rigid or entrenched in their opposing roles [42], the approach of the complementary partnership we describe is one of flexibility, adapting support to best suit the dynamic demands of therapy activities and the child’s need for support.

### Parent Beliefs About and Attitudes Towards Therapy

The parent beliefs and attitudes towards therapy reflect an eagerness to persevere when therapy is not easy, critical thinking about how to integrate therapy in their family context, and flexibility in changing their expectations or approaches as therapy progresses. Parents who are committed, think critically about therapy demands, and bring an attitude of flexibility to therapy may be in the “active collaborative mode,” in that they believe they have an active part in the change process, are open in disclosing issues with the therapist, and take initiative to continue the work [43]. It is also possible that a parent’s therapy criticalness might lead them to enter either of the two other collaborative modes, mutual (i.e., joint collaboration where both the client and therapist contribute to the change process) or dependent (i.e., the client relies on the therapist to be the main contributor to the change process) [43]. For example, if the parent tells the therapist they are having difficulties with implementing a coping strategy at home, the therapist can collaborate with the client in making appropriate changes to the approach (i.e., mutual collaborative mode), but if the parent thinks that home practice is too difficult to implement given their family’s situation, then they may rely on the therapist to take a greater role in the change process (i.e., dependent collaborative mode).

### Limitations

This study has several limitations. Parent therapeutic functions were only examined within the context of a manualized child CBT program, and it is unclear if similar therapeutic factors would emerge or be as relevant in other treatment modalities (e.g., play therapy, emotion focused therapy, family therapy). Some specific ways that parents supported their children in therapy are unique to CBT approaches, such as CBT’s emphasis on skill learning and home practice (which then necessitates parents’ support with facilitating and reminding the child to complete home practice). Given that this study focused on parent involvement in therapy targeting emotion regulation, it is difficult to determine the extent to which these findings might generalize to treatment focusing on other more specific presenting concerns. The importance of parent involvement in treatment for child anxiety, OCD, and behaviour problems is well-documented in the literature (e.g., [9, 11, 15–17]), and it is possible that the noted parent therapeutic functions may apply to the treatment of these specific conditions to varying degrees. For example, the “co-facilitator” function would likely be of paramount importance in treatment for OCD, which typically requires consistent exposure and response prevention practice outside of the therapy session. On the other hand, the “companion and teammate” and “coach and cheerleader” functions are highly aligned with evidence-based parenting strategies for behaviour problems (e.g., positive praise, play, rewards). Further research is needed to examine how the findings from this study may generalize to mental health treatments for other presenting concerns in autistic children.

This sample was only comprised of parents of autistic children, and the parent therapeutic factors that emerged from this study may not be generalizable to child therapy more broadly. At the same time, many of the factors may also be useful for children with other neurodevelopmental conditions. For example, a child with ADHD and challenges with executive functions (e.g., planning and organization) may benefit from a parent providing support as the logistical coordinator, co-facilitator, and/or the complementary helper. There was also a substantial delay between participants’ final *SAS:OR* session and their participation in the current study which may have affected their ability to remember details of their involvement in treatment.

There were also limitations in the demographics and characteristics of the sample. This sample only included parents of school-aged children, and parent involvement would likely differ in numerous ways as children transition to adolescence; prior research has discussed relevant considerations for parents in the treatment of anxiety symptoms for autistic adolescents [5]. Participants were predominantly female, and there was a lack of ethnic or cultural diversity (the majority of parents were White). Further research is needed to explore the ways in which gender or culture might influence the parent therapeutic factors identified in this study. For example, a recent study found that compared to White parents, those who identified as Latinx were less likely to share their concerns about treatment with therapists and tended to have lower levels of participation in sessions [8].

## Summary

Overall, this study identified a number of clinically relevant constructs and factors associated with successful operations in parent-involved child mental health treatment, and outlined a conceptual framework that can serve as a guide for further empirical study. It brings to light many of the otherwise implicit demands that can be placed on parents in their contributing roles to help their children, and it is important to consider how different families will have unique capabilities and resources to dedicate to this kind of endeavor. To further our understanding of parent-involved therapy, future work should seek to develop measures of parent therapeutic factors. An observational, interview-based, or questionnaire-type measure would also enable an investigation of the relation between parent therapeutic factors and child treatment outcomes. This would allow for the empirical testing of the conceptual framework and provide targets for clinicians to improve parent involvement and inform decisions about which families might benefit from interventions that expect parent involvement.

## Electronic Supplementary Material

Below is the link to the electronic supplementary material.

Supplementary Material 1

## Data Availability

The data supporting the findings of this study will be made available upon reasonable request for academic use and within the limitations of the provided informed consent. All requests for raw and analyzed data will be reviewed by York University’s research ethics committee to verify if the request is subject to any participant confidentiality obligations.

## References

[1] Sukhodolsky DG, Bloch MH, Panza KE, Reichow B (2013) Cognitive-behavioral therapy for anxiety in children with high-functioning autism: a meta-analysis. Pediatrics 132:e1341–e1350 24167175 10.1542/peds.2013-1193 PMC3813396

[2] van Steensel FJA, Bögels SM (2015) CBT for anxiety disorders in children with and without autism spectrum disorders. J Consult Clin Psychol 83:512–523 25894668 10.1037/a0039108

[3] Wood JJ, Kendall PC, Wood KS, Kerns CM, Seltzer M, Small BJ et al (2020) Cognitive behavioral treatments for anxiety in children with autism spectrum disorder: A randomized clinical trial. JAMA Psychiatry 77:474–483 31755906 10.1001/jamapsychiatry.2019.4160 PMC6902190

[4] Moree BN, Davis TE (2010) Cognitive-behavioral therapy for anxiety in children diagnosed with autism spectrum disorders: Modification trends. Res Autism Spectr Disord 4:346–354

[5] Reaven J (2011) The treatment of anxiety symptoms in youth with high-functioning autism spectrum disorders: Developmental considerations for parents. Brain Res 1380:255–263 20875799 10.1016/j.brainres.2010.09.075

[6] Ehrenreich-May J, Storch EA, Queen AH, Hernandez Rodriguez J, Ghilain CS, Alessandri M et al (2014) An open trial of cognitive-behavioral therapy for anxiety disorders in adolescents with autism spectrum disorders. Focus Autism Other Dev Disabl 29:145–155

[7] Wood JJ, Ehrenreich-May J, Alessandri M, Fujii C, Renno P, Laugeson E et al (2015) Cognitive behavioral therapy for early adolescents with autism spectrum disorders and clinical anxiety: A randomized, controlled trial. Behav Ther 46:7–19 25526831 10.1016/j.beth.2014.01.002 PMC4272761

[8] Guan K, Lau AS, Zhang A, Chlebowski C, Haine-Schlagel R, Brookman-Frazee L (2019) In-session caregiver behaviors during evidence-based intervention delivery for children with ASD in community mental health services. Evid Based Pract Child and Adolesc Ment Health 4:55–71 10.1080/23794925.2019.1565500 PMC6602542 31263772

[9] Perihan C, Burke M, Bowman-Perrott L, Bicer A, Gallup J, Thompson J et al (2020) Effects of cognitive behavioral therapy for reducing anxiety in children with high functioning ASD: A systematic review and meta-analysis. J Autism Dev Disord 50:1958–1972 30810842 10.1007/s10803-019-03949-7

[10] Dickson KS, Chlebowski C, Haine-Schlagel R, Ganger B, Brookman-Frazee L (2022) Impact of therapist training on parent attendance in mental health services for children with ASD. J Clin Child Adolesc Psychol 51:230–241 32816564 10.1080/15374416.2020.1796682 PMC7987108

[11] Norman KR, Silverman WK, Lebowitz ER (2015) Family accommodation of child and adolescent anxiety: Mechanisms, assessment, and treatment. J Child Adolesc Psychiatr Nurs 28:131–140 26238937 10.1111/jcap.12116 PMC4896065

[12] Anderson S, Morris J (2006) Cognitive behaviour therapy for people with Asperger syndrome. Behav Cogn Psychother 34:293–303

[13] Griffin C, Lombardo MV, Auyeung B (2016) Alexithymia in children with and without autism spectrum disorders. Autism Res 9:773–780 26426084 10.1002/aur.1569

[14] Corbett BA, Constantine LJ, Hendren R, Rocke D, Ozonoff S (2009) Examining executive functioning in children with autism spectrum disorder, attention deficit hyperactivity disorder and typical development. Psychiatry Res 166:210–222 19285351 10.1016/j.psychres.2008.02.005 PMC2683039

[15] Sofronoff K, Attwood T, Hinton S (2005) A randomised controlled trial of a CBT intervention for anxiety in children with Asperger syndrome. J Child Psychol Psychiatry 46:1152–1160 16238662 10.1111/j.1469-7610.2005.00411.x

[16] Thomson K, Burnham Riosa P, Weiss JA (2015) Brief report of preliminary outcomes of an emotion regulation intervention for children with autism spectrum disorder. J Autism Dev Disord 45:3487–3495 25877014 10.1007/s10803-015-2446-1

[17] Stadnick N, Drahota A, Brookman-Frazee L (2013) Parent perspectives of an evidence-based intervention for children with autism served in community mental health clinics. J Child Fam Stud 22:414–422 24019736 10.1007/s10826-012-9594-0 PMC3765032

[18] Kendall PC (2011) Child and adolescent therapy, fourth edition: Cognitive-behavioral procedures. Guilford Press, New York NY

[19] Hoffman C, Crnic KA, Baker JK (2006) Maternal depression and parenting: Implications for children’s emergent emotion regulation and behavioral functioning. Parent Sci Pract 6:271–295

[20] Ting V, Weiss JA (2017) Emotion regulation and parent co-regulation in children with autism spectrum disorder. J Autism Dev Disord 47:680–689 28070784 10.1007/s10803-016-3009-9 PMC5352765

[21] Gulsrud AC, Jahromi LB, Kasari C (2010) The co-regulation of emotions between mothers and their children with autism. J Autism Dev Disord 40:227–237 19714458 10.1007/s10803-009-0861-x PMC2810360

[22] Hougaard E (1994) The therapeutic alliance—A conceptual analysis. Scand J Psychol 35:67–85 8191262 10.1111/j.1467-9450.1994.tb00934.x

[23] Hock R, Yingling ME, Kinsman A (2015) A parent-informed framework of treatment engagement in group-based interventions. J Child Fam Stud 24:3372–3382

[24] Solish A, Perry A (2008) Parents’ involvement in their children’s behavioral intervention programs: Parent and therapist perspectives. Res Autism Spectr Disord 2:728–738

[25] Palinkas LA, Horwitz SM, Green CA, Wisdom JP, Duan N, Hoagwood K (2015) Purposeful sampling for qualitative data collection and analysis in mixed method implementation research. Adm Policy Ment Health 42:533–544 24193818 10.1007/s10488-013-0528-y PMC4012002

[26] Weiss JA, Thomson K, Burnham Riosa P, Albaum C, Chan V, Maughan A et al (2018) A randomized waitlist-controlled trial of cognitive behavior therapy to improve emotion regulation in children with autism. J Child Psychol Psychiatry 59:1180–1191 29687457 10.1111/jcpp.12915 PMC6220772

[27] Corbin JM, Strauss A (2008) Basics of qualitative research: Techniques and procedures for developing grounded theory, 3rd edn. SAGE Publications, Thousand Oaks CA

[28] Charmaz K (2006) Constructing grounded theory: A practical guide through qualitative analysis. SAGE Publications, Thousand Oaks CA

[29] Engward H (2013) Understanding grounded theory. Nurs Stand 28:37–41 24128248 10.7748/ns2013.10.28.7.37.e7806

[30] Maughan AL, Weiss JA (2017) Parental outcomes following participation in cognitive behavior therapy for children with autism spectrum disorder. J Autism Dev Disord 47:3166–3179 28762160 10.1007/s10803-017-3224-z

[31] Safran JD, Segal ZV, Vallis TM, Shaw BF, Samstag LW (1993) Assessing patient suitability for short-term cognitive therapy with an interpersonal focus. Cognit Ther Res 17:23–38

[32] Sameroff AJ, Fiese BH (2000) Transactional regulation: The developmental ecology of early intervention. In: Shonkoff JP, Meisels SJ (eds) Handbook of early childhood intervention, vol 2. Cambridge Universities Press, New York NY, pp 135–159

[33] Woodman AC, Mawdsley HP, Hauser-Cram P (2015) Parenting stress and child behavior problems within families of children with developmental disabilities: Transactional relations across 15 years. Res Dev Disabil 36:264–276 10.1016/j.ridd.2014.10.011 PMC4425632 25462487

[34] Eyberg SM, Robinson EA (1982) Parent-child interaction training: Effects on family functioning. J Clin Child Psychol 11:130–137

[35] Bronfenbrenner U, Morris PA (2007) The bioecological model of human development. In: Lerner RM (ed) Handbook of child psychology, vol 1. John Wiley & Sons, New York NY, pp 793–828

[36] Rao PA, Beidel DC (2009) The impact of children with high-functioning autism on parental stress, sibling adjustment, and family functioning. Behav Modif 33:437–451 19436073 10.1177/0145445509336427

[37] Dunn ME, Burbine T, Bowers CA, Tantleff-Dunn S (2001) Moderators of stress in parents of children with autism. Community Ment Health J 37:39–52 11300666 10.1023/a:1026592305436

[38] Nevill RE, Lecavalier L, Stratis EA (2018) Meta-analysis of parent-mediated interventions for young children with autism spectrum disorder. Autism 22:84–98 29490483 10.1177/1362361316677838

[39] Vygotsky LS (1978) Mind in Society: The development of higher psychological processes. Harvard University Press, Cambridge MA

[40] Goldfried MR, Burckell LA, Eubanks-Carter C (2003) Therapist self-disclosure in cognitive-behavior therapy. J Clin Psychol 59:555–568 12696131 10.1002/jclp.10159

[41] Patterson J, Williams L, Edwards TM, Chamow L, Grauf-Grounds C (2009) Working with couples. In: Nichols MP (ed) Essential skills in Family Therapy: From the first interview to termination, 2nd edn. Guilford Press, New York NY, pp 160–183

[42] Burnham JB (2002) Family Therapy: First steps towards a systemic approach. Routledge, London UK

[43] Bachelor A, Laverdière O, Gamache D, Bordeleau V (2007) Clients’ collaboration in therapy: Self-perceptions and relationships with client psychological functioning, interpersonal relations, and motivation. Psychother (Chic) 44:175–192 10.1037/0033-3204.44.2.175 22122209

