# Supplementary material for: Parent Involvement in Mental Health Treatment for Autistic Children: A Grounded Theory-Informed Qualitative Analysis
Source: Child Psychiatry Hum Dev. 2023 Oct 17;56(4):982–95. doi: 10.1007/s10578-023-01621-x (PMC12289757; doi:10.1007/s10578-023-01621-x)
Supplement: Supplementary file 1 — Supplementary Material 1 [file 10578_2023_1621_MOESM1_ESM.docx]

**Appendix A**

***Parent Interview Guide***

**Appendix B**

***Therapist Interview Guide***
